# Case report of first-episode psychotic symptoms in a patient with early-onset Alzheimer’s disease

**DOI:** 10.1186/s12888-020-02537-9

**Published:** 2020-03-17

**Authors:** Xiao Li, Zhenzhen Xiong, Yaya Liu, Yiwen Yuan, Junfeng Deng, Weiyi Xiang, Zhe Li

**Affiliations:** 1 Mental Health Center and National Clinical Research Center for Geriatrics, West China Hospital, Sichuan University, No. 28 Dian Xin Nan Road, Chengdu, 610041 Sichuan China; 2 School of Nursing, Chengdu Medical College, Chengdu, 610083 Sichuan China; 3 Zun Yi Psychiatric Hospital, Zunyi, 563000 Guizhou China; 4 The West China College of Medicine, Sichuan University, Chengdu, 610041 Sichuan China

**Keywords:** Brain imaging, Cognitive function, Dementia, Schizophrenia, SORL1

## Abstract

**Background:**

Alzheimer’s disease (AD) is a neurodegenerative disorder featuring the behavioral and psychological symptoms of dementia. Patients with early-onset AD that exhibits first as psychotic symptoms usually lack obvious cognitive impairment, so they may be misdiagnosed with late-onset schizophrenia.

**Case presentation:**

We report a patient who had prominent psychotic symptoms at the age of 60 and was initially diagnosed with very-late-onset-schizophrenia-like psychosis. Psychotic symptoms disappeared rapidly after treatment with olanzapine, and the patient later showed extrapyramidal symptoms and decline in cognitive function. Brain magnetic resonance imaging (MRI) showed frontotemporal atrophy, and positron emission tomography (PET) showed extensive areas of hypometabolism in the frontal cortex and head of the caudate nucleus. The patient’s *SORL1* gene was found to carry a heterozygrous mutation (c.296A > G). The patient was eventually diagnosed with early-onset AD.

**Conclusions:**

Our case suggests that clinicians should consider the possibility of early-onset AD in middle-aged or elderly patients whose first symptoms are the behavioral and psychological symptoms of dementia. To distinguish early-onset AD from late-onset schizophrenia, clinicians should evaluate cognitive function, perform MRI and PET, and search for *SORL1* mutations.

## Background

Alzheimer’s disease (AD) is a common neurodegenerative disorder characterized by progressive cognitive impairment [1, 2]. In 2010, AD affected 4.7 million individuals in the US alone and by 2050 an estimated 13.8 million people may have AD in the US [3]. It was reported that the prevalence of AD is 3.21% among individuals aged 65 years and older in China [4], which poses a huge challenge and burden for elderly, families, caregivers and wider society. Because AD is chronic, early identification and intervention are important to improve prognosis.

AD is typically classified as early-onset if it occurs before the age of 65, or late-onset if it occurs later [5]. The early-onset form of the disease accounts for only 5.5% of all AD cases, so it is often overlooked [6]. The behavioral and psychological symptoms of dementia (BPSD) including agitation, aggression, apathy, depression, psychosis, disinhibition can occur early in AD, even before the onset of dementia [7, 8]. It can be challenging to distinguish between BPSD in AD and other mental disorders, such as major depressive disorder or schizophrenia, especially very late-onset schizophrenia-like psychosis (VLOSLP). This type of psychosis, which shows a community prevalence of 0.1–0.5% [9], generally occurs after the age of 60 and is characterized by psychotic symptoms such as delusions and hallucinations. Patients with VLOSLP usually have psychotic symptoms and mild cognitive deficits, so they may be misdiagnosed as having dementia [10]. Patients in early stages of AD may manifest only behavioral and psychological symptoms of dementia without obvious decline in cognitive function, so they may be misdiagnosed with VLOSLP.

Mutations in the *SORL1* gene, which encodes a protein involved in the processing of amyloid-beta (Aβ) precursor protein and in secretion of the Aβ peptide, have been associated with AD [11]. The potential role of these mutations in the disease has been neglected in favor of studies of much more frequent mutations in such genes as *APP, PSEN1* and *PSEN2* [12] .

Here we present a patient with AD and *SORL1* mutations whose first-episode psychotic symptoms occurred before the age of 65 and who was initially misdiagnosed with schizophrenia. To the best of our knowledge, this is the first report of a patient with early-onset AD whose first symptoms were psychotic and extrapyramidal.

## Case presentation

A right-handed, 63-year-old Chinese woman, with 12 years of education, was admitted to our hospital in September 2018 at the request of her family members.

### History of presenting complaint

In 2016, the patient had developed psychotic symptoms, including delusions and auditory hallucination, without an apparent cause. She said someone wanted to harm her and was talking about her. Sometimes she told her family members that she heard knocking on the door, and she exhibited diminished emotional expression and avolition. She required the support of family members to conduct daily activities and communicate with others. Her sleep quality was poor, but she showed no disturbance of consciousness. She did not seek medical advice and receive any remedy at that time.

**Fig. 1:**
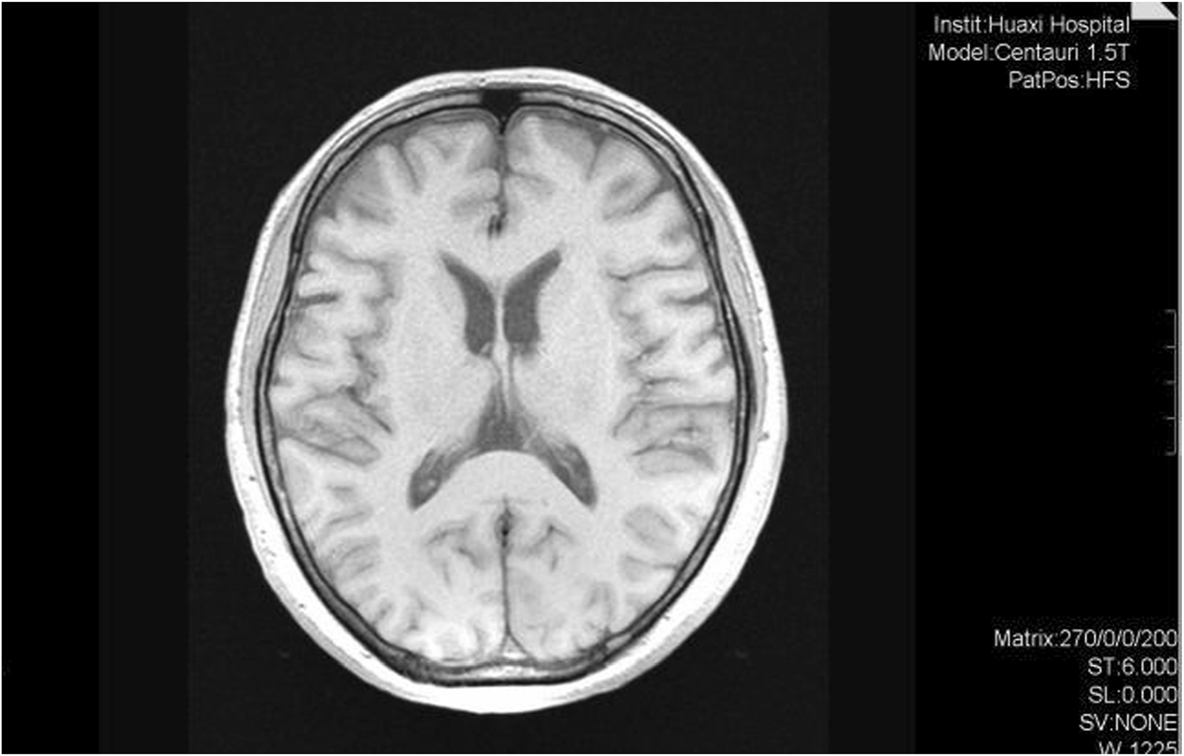
Brain MRI in 2016. No structural abnormality has been found

After these symptoms had continued for about 12 months, she was taken by her family to the outpatient department of our hospital. Brain magnetic resonance imaging (MRI) did not reveal any obvious structural abnormalities (Fig. 1). She was diagnosed with schizophrenia by an experienced psychiatrist based on criteria in the fifth edition of the Diagnostic and Statistical Manual of Mental Disorders [13]. Delusions and hallucinations disappeared after the patient took 5 mg of olanzapine every night for 1 month. We monitored the blood glucose, blood lipid, blood pressure and weight every month to investigate the possibility of metabolic syndrome after treatment with olanzapine. However, she began to walk slowly, her neck became rigid, and she experienced episodic memory impairment, leading her to forget new events, ask the same question repeatedly and get lost in new places. Her physical functions, such as dressing and eating, were also impaired. However, she did not develop language dysfunction, and she was able to communicate smoothly with family members. She was treated with benzhexol hydrochloride at 2 mg every night for extrapyramidal symptoms, but the symptoms did not improve. The daily dose of olanzapine was reduced to 2.5 mg in July 2017, but the symptoms still did not improve after 1 month. Therefore, we believe that her symptoms did not cause by olanzapine then stop using benzhexol hydrochloride. In May 2018, her neck stiffness became worse and gradually spread to the limbs. She developed severe neck, limb and postural rigidity, but no tremor. She walked unstably, exhibited bradykinesia and memory decline, and showed obvious decline in her ability to take care of herself. On the other hand, she did not exhibit socially inappropriate behaviors, apathy, or dietary changes, and she responded normally to family members’ feelings.

At her admission to our hospital in September 2018, the patient reported never smoking or drinking. She had previously undergone gallbladder surgery. She was in good health and had no family history of mental disorders. The patient had good support from family members and did not report any major adverse life events. The patient and her family members denied any drug abuse. She did not have any history of violence, agitation and suicidal behavior during the course of her illness. Her vital signs were stable, and no abnormal physical signs were detected at admission. Blood and urine tests, blood glucose level, liver and renal function and thyroid function were normal, and the patient showed no evidence of infection. Electroencephalography, electrocardiography, and transcranial doppler ultrasound results were normal. Mental status examination showed no signs of hallucinations or delusions. The patient had stable mood but temporal disorientation and deficits in attention, calculation and language competence, as well as delayed recall. She also exhibited impairments in visuospatial organization and abstract thinking. She had no insight into her disease. The total score on the MMSE was 16 and on the MoCA was 9.

**Fig. 2:**
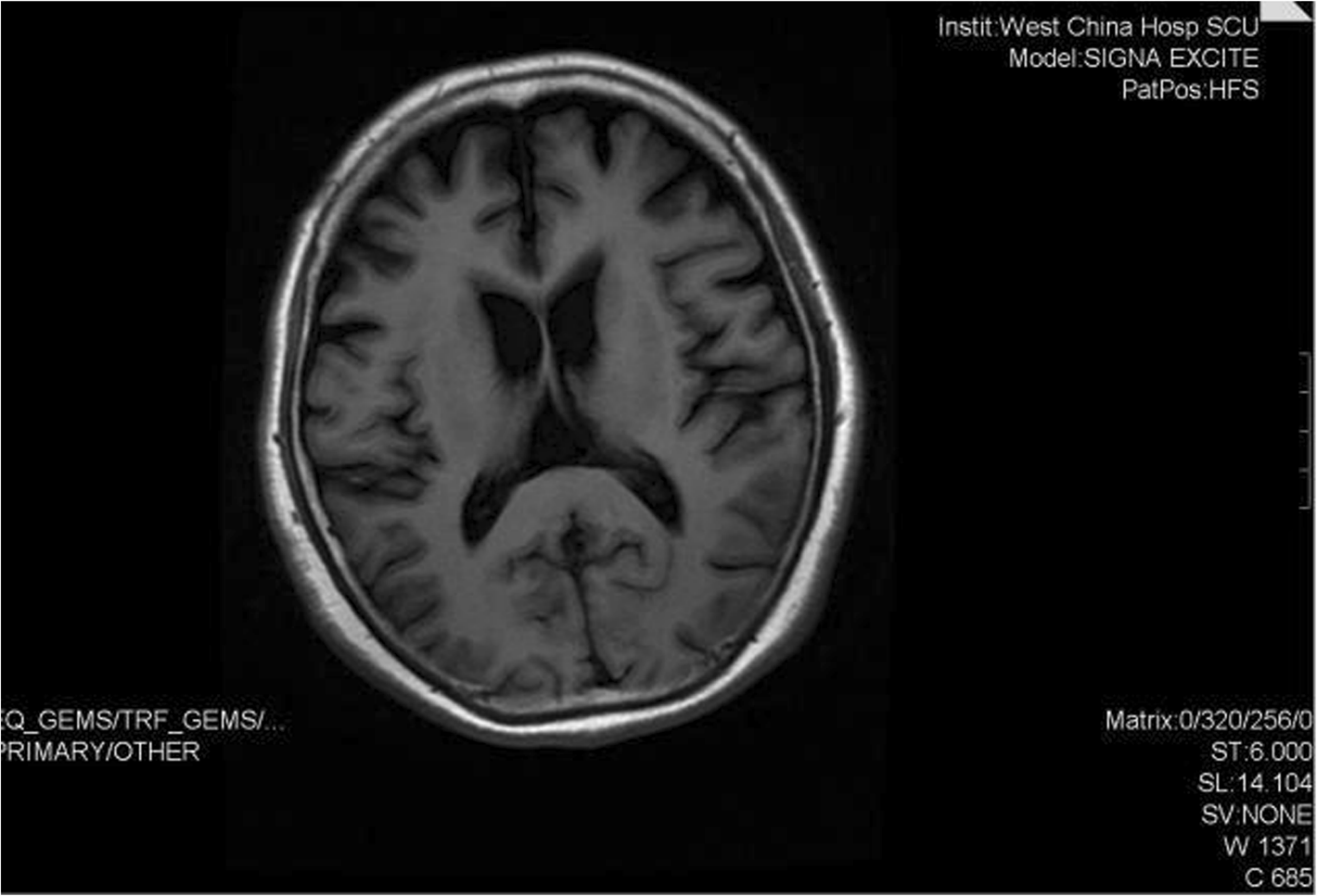
The brain MRI in September 2018. Brain MRI showed mild bilateral frontotemporal atrophy

During hospitalization, brain MRI showed mild frontotemporal atrophy relative to the MRI in 2016 (Fig. 2). She scored 16/30 on the Mini-Mental State Exam (MMSE). She exhibited temporal disorientation and deficits in attention, calculation and language competence, as well as delayed recall. She scored 9/30 on the Montreal Cognitive Assessment (MoCA), indicating impairments in visuospatial organization, attention, language ability, temporal orientation, and abstract thinking. Her total score on the Activity of Daily Living Scale (ADL) [14] was 54, indicating difficulty in performing daily life activities (Table 1). Based on her two-year history of using antipsychotics and hypermyotonia, she was diagnosed with tardive dyskinesia. After consultation with experts in the neurology department, her diagnosis was changed to Parkinson’s disease on the basis of her bradykinesia and hypermyotonia. Olanzapine treatment was replaced with the combination of levodopa and benserazide hydrochloride (375 mg/d), pramipexole hydrochloride (0.75 mg/d), Selegiline hydrochloride (5 mg/d), and benzhexol hydrochloride (3 mg/d). Her bradykinesia, rigidity and shuffling gait improved after 2 weeks, but not her memory. She was discharged in October 2018.

**Table 1:** Scores of Activity of Daily Living (ADL) scale in 2018

Item	Score	Item	Score
Transportation	4	Personal hygiene	4
Walking	4	Washing	4
Food preparation	4	Bath/shower	4
Housework	4	Shopping	4
Medication	3	Toilet/commode	4
Eating	3	Telephone	4
Dressing	4	Finances	4

Note: Each item gets 1 point for normal. The score of 2–4 points represent function decline in each item

### Follow up/reviews

**Fig. 3:**
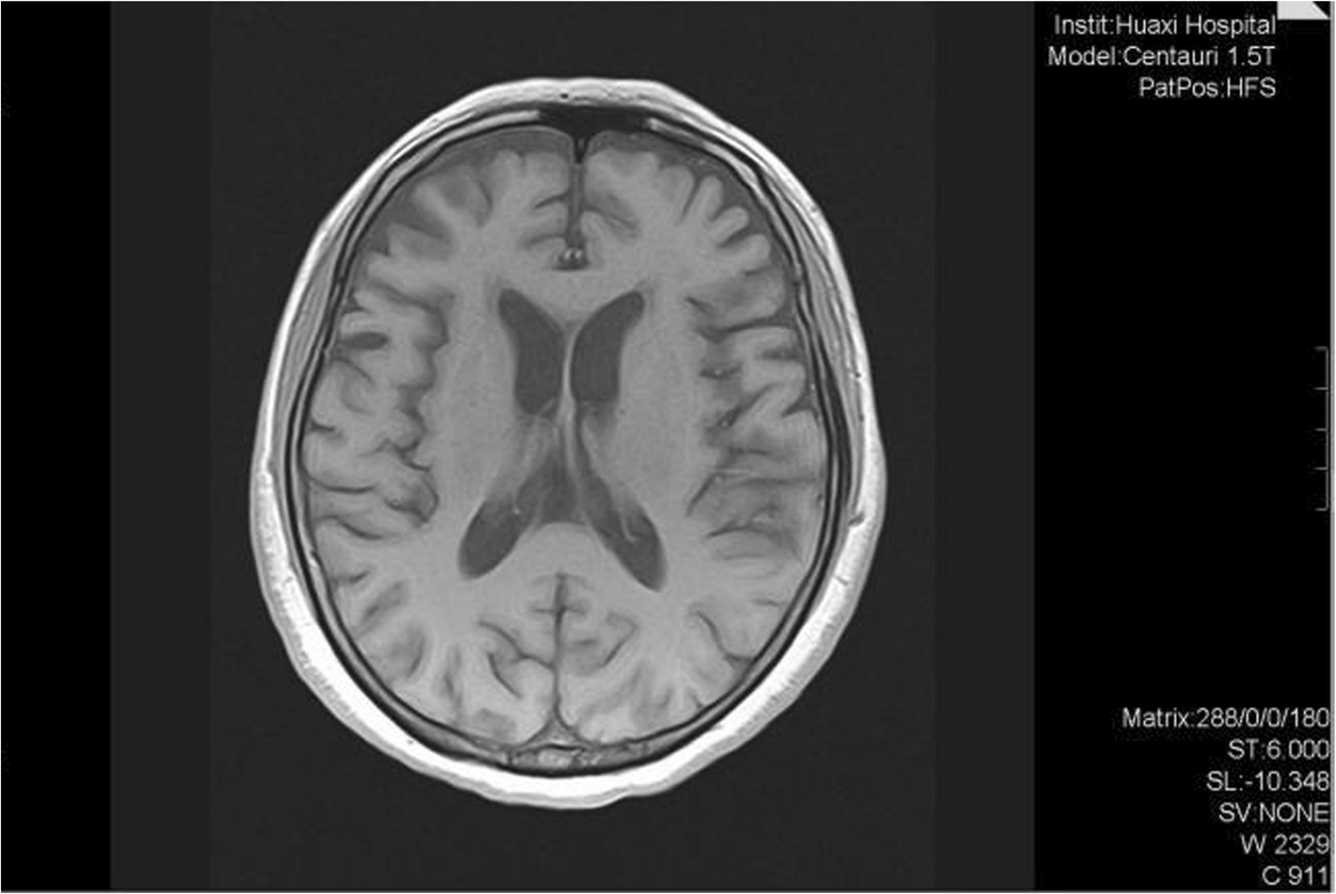
The brain MRI in August 2019. Brain MRI Showed moderate bilateral frontotemporal atrophy

**Fig. 4:**
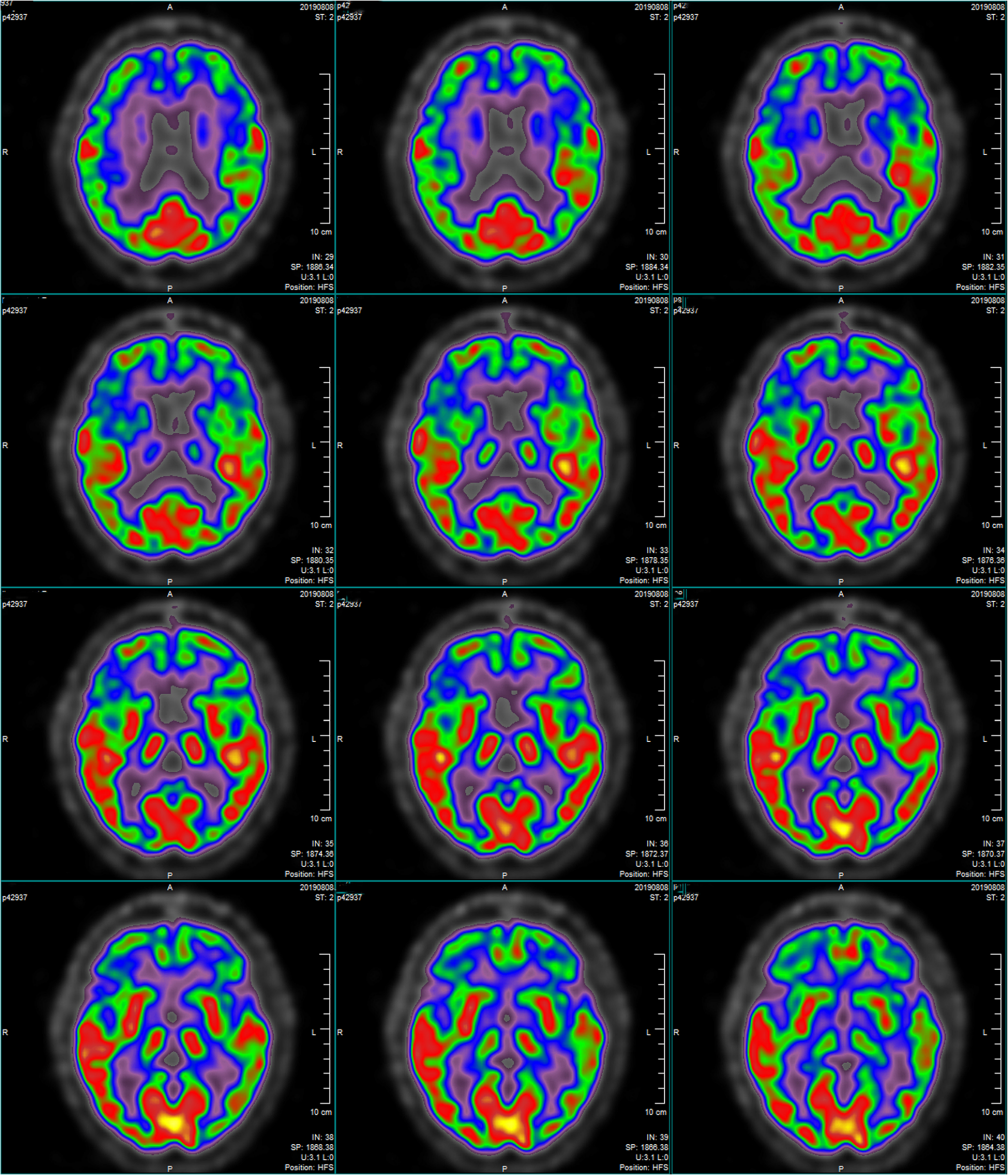
The 18F-FDG PET/CT in August 2019. Extensive areas of hypometabolism in frontal cortex and head of caudate nucleus

During a follow-up visit 2 months after discharge, 53 genes associated with dementia were analyzed (Table 2). The only mutation detected was a heterozygous variant in *SORL1* (chr11:121340726, c.296A > G). She showed a higher score of 20/30 on both the MMSE and MoCA. She was prescribed with memantine (10 mg/d) and donepezil (10 mg/d) and stopped taking benzhexol hydrochloride, but her cognitive function showed no change at follow-up in July 2019. At follow-up in August 2019, brain MRI detected moderate frontotemporal atrophy that appeared more serious than in 2018 (Fig. 3), while ^18^F-fluorodeoxyglucose-based positron emission tomography/computed tomography demonstrated bilateral frontal and caudate hypometabolism (Fig. 4). The score on MoCA improved to 23/30, but the score on the ADLS fell to 31. She was unable to complete many daily life activities (Table 3).

**Table 2:** A list of genes analyzed

Gene Analyzed							
A2M	ACE	ACHE	ADAM10	APBB2	APOE	APP	ATN1
ATP13A2	BCHE	C9orf72	CHMP2B	CHRM1	CLU	CR1	DNMT1
FAM134B	GBA	GRN	GSTO1	HFE	HNRNPA1	HNRNPA2B1	ITM2B
KLK1	LOC643387	MAPT	MEOX2	MPO	NOS3	NOTCH3	NPC1
PAXIP1	PICALM	PLAU	PLD3	PRNP	PSEN1	PSEN2	RPS27A
SLC6A4	SNCA	SNCB	SORL1	SQSTM1	TARDBP	TBK1	TNFSF14
TREM2	TRPM7	TYROBP	UBQLN2	VCP

**Table 3:** Scores of Activity of Daily Living (ADL) scale in 2019

Item	Score	Item	Score
Transportation	3	Personal hygiene	1
Walking	3	Washing	3
Food preparation	2	Bath/shower	3
Housework	3	Shopping	2
Medication	3	Toilet/commode	2
Eating	1	Telephone	1
Dressing	3	Finances	1

Note: Each item gets 1 point for normal. The score of 2–4 points represent function decline in each item

Our patient was diagnosed with probable AD because of early, significant episodic memory impairment, medial temporal lobe atrophy, brain glucose hypometabolism based on PET and AD-associated genetic mutation [15]. She was prescribed memantine (10 mg/d) and donepezil (10 mg/d). At her last follow-up in October 2019, the patient showed poor memory, slow reaction and bradykinesia, but no psychotic symptoms.

### Diagnostic symptoms

#### Alzheimer’s disease

**Fig. 5:**
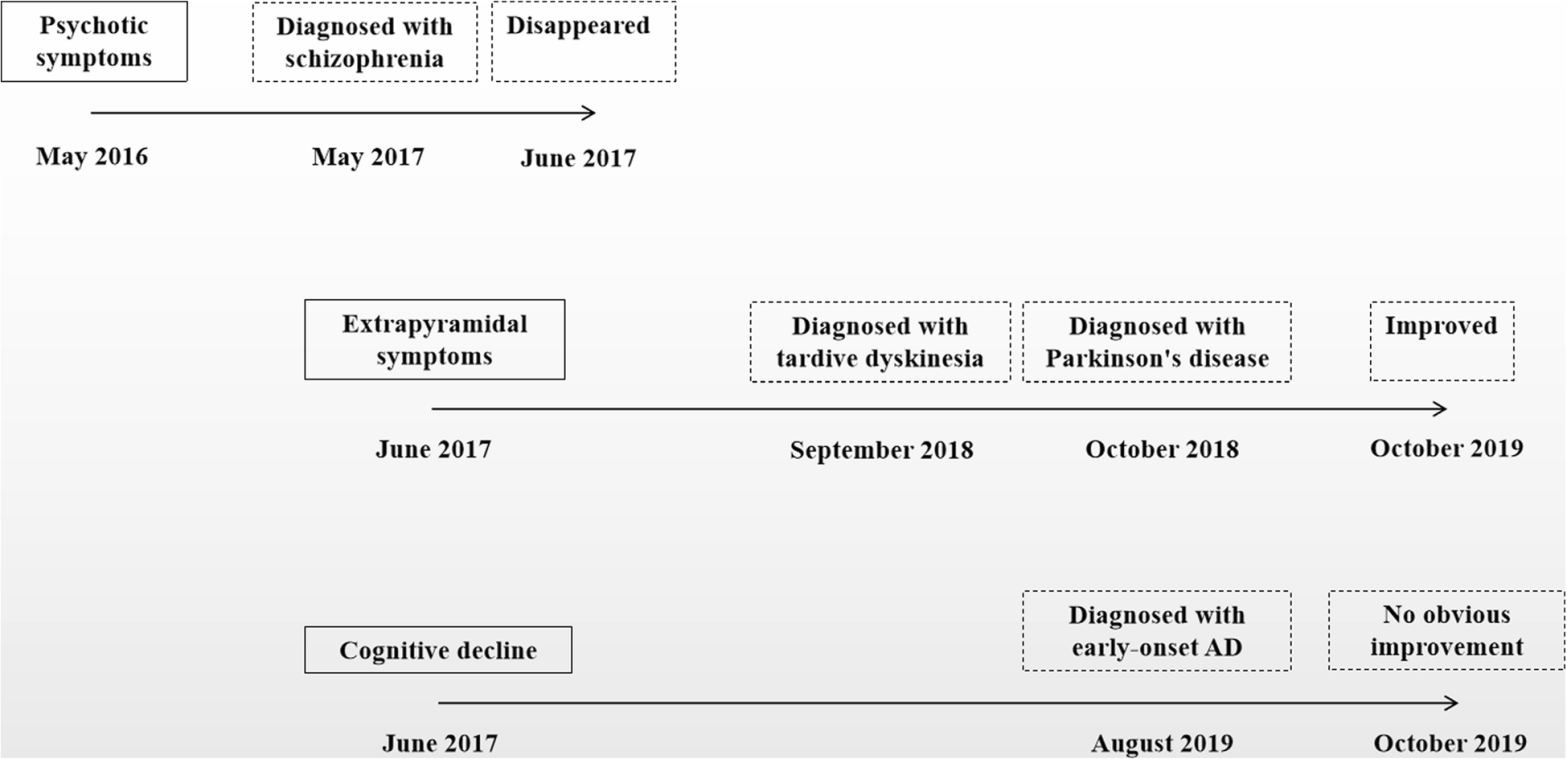
Medical history of the patient

The patient had temporal disorientation and deficits in attention, calculation and language competence, significant episodic memory impairment. She had significant temporal lobe atrophy, brain glucose hypometabolism based on PET and AD-associated genetic mutation. A timeline of the historical and current information is shown in Fig. 5.

### Differential diagnostic symptoms

#### VLOSLP

The psychotic symptoms of the patient occur after the age of 60, but disappeared after treatment with antipsychotics in 1 month. She did not display the clinical course typical of patients with schizophrenia. Frontotemporal dementia: The patient did not display the impaired language function typical of semantic variant primary progressive aphasia or the non-fluent/agrammatic variant of primary progressive aphasia [16, 17]. She also did not display early behavioral disinhibition, apathy, loss of sympathy or empathy, dietary changes, or deficits in executive tasks typical of the behavioral variant of frontotemporal dementia [18].

## Discussion and conclusions

To the best of our knowledge, this is the first report of an AD patient with mutations in the SORL1 gene who presented initially psychotic symptoms before 65 years old, but she was misdiagnosed with schizophrenia. 1 month after treatment with antipsychotics, her psychotic symptoms disappeared, but she began to experience rigidity, bradykinesia, and impaired cognitive function. She did not display the clinical course typical of patients with schizophrenia. These considerations, combined with results from neuropsychological examinations, brain MRI, fluorodeoxyglucose PET and gene mutation analysis, led us to diagnose the patient with early-onset AD.

AD is the most common cause of dementia and is characterized by the presence of Aβ plaques and tau-containing neurofibrillary tangles in the brain [19]. Many AD patients eventually display behavioral and psychological symptoms of dementia, which include four neuropsychiatric sub-symptoms: hyperactivity, psychosis, affective symptoms and apathy [20]. A population-based study of Chinese elderly with dementia found that half had at least one behavioral or psychological symptom of dementia [21]. Psychiatric symptoms are commonly seen in the early stage of dementia and can be present at all stages of disease, but rarely seen in early-onset AD [22, 23]. Behavioral and psychological symptoms of dementia in AD can increase the cost of health care, reduce quality of life, lead to long-term hospitalization and place an enormous burden on caregivers [24]. Therefore, early identification and treatment of such symptoms can help improve the prognosis of AD patients.

The patient experienced the symptoms of rigidity, bradykinesia and cognitive decline after olanzapine treatment. Although olanzapine can cause extrapyramidal symptoms such as those observed in our patient [25], we believe that the drug was not the cause in our patient. Olanzapine is associated with lower incidence of such symptoms than first-generation antipsychotics [26], and the symptoms in our patient did not improve when the olanzapine dose was reduced or when the drug was replaced with benzhexol hydrochloride. We hypothesize that the extrapyramidal symptoms in our patient were part of the manifestations of AD with parkinsonian symptoms. These symptoms are observed in many patients with AD, and they are associated with reduced ability to perform daily activities and with severe cognitive impairment, including attention and visuospatial deficit [27, 28]. In a longitudinal study has also found that progressive worsening of parkinsonism is strongly associated with cognitive decline in AD patients [29]. Screening for such symptoms may allow early detection of AD, and these symptoms may be useful as a prognostic marker for predicting cognitive function [28]. In early AD, extrapyramidal symptoms have also been associated with pathological changes in substantia nigra, including α-synuclein aggregation, hyperphosphorylated tau accumulation, and neuronal loss [30].

Our previous work has linked the following 21 well-studied genes with AD: *TGFB1, CTNNB1, APP, IL1B, PSEN1, PTGS2, IL6, VEGFA, SOD1, AKT1, CDK5, TNF, GSK3B, TP53, CCL2, BDNF, NGF, IGF1, SIRT1, AGER* and *TLR* [31]. Other work has also linked mutations in *APP, PSEN1* and *PSEN2* with early-onset AD [12]. Although we analyzed 53 genes associated with dementia, our patient showed a mutation only in the *SORL1* gene. The *SORL1* gene encodes a protein involved in the processing of amyloid-beta (Aβ) precursor protein and in secretion of the Aβ peptide. The SORL1 has been found to be associated with age-related cognitive decline in episodic memory and processing speed in non-demented older adults [32]. The mutations in this gene have also been reported in AD, they appear to play a critical role in the disease [11] and may even be a risk factor for the early-onset form [33], consistent with the role of *SORL1* in Aβ processing and secretion. Therefore, the early cognitive decline of our patients including temporal disorientation, deficits in attention, calculation and language competence, significant episodic memory impairment may associate with the mutations in this gene.

The previous study indicated that the mutations in SORL1 gene are associated with parkinsonian features in AD patients, such as tremor at rest, hypophonia, micrographia, masked facial expression, smaller steps on gait, and overall bradykinesia [34]. In addition, the SORL1 variations are found to influence the brain structure. A structural MRI study in Chinese population has found the SORL1 variants are associated with grey matter volume (GMV) reduction of the right middle temporal pole in older adults [32]. Another brain structural study in Chinese population has found individuals carrying SORL1 allele A showing reduced grey matter volumes in the right posterior cingulate, left middle occipital, medial frontal, and superior temporal gyri [35]. These results suggested that the SORL1 variants have an effect on brain structure. Our patient had frontotemporal lobe atrophy, this brain structural alterations might associate with SORL1 gene mutation. Therefore, the SORL1 gene mutation could lead to the development and progression of cognitive decline, parkinsonian motor symptoms and brain structural alterations. Screening for SORL1 mutations may be useful for patients suspected of having early-onset AD.

Our patient had frontotemporal lobe atrophy, but not frontotemporal dementia. She did not display the impaired language function typical of semantic variant primary progressive aphasia or the non-fluent/agrammatic variant of primary progressive aphasia [16, 17]. She also did not display early behavioral disinhibition, apathy, loss of sympathy or empathy, dietary changes, or deficits in executive tasks typical of the behavioral variant of frontotemporal dementia [18]. These considerations, combined with the fact that *SORL1* mutation has not been associated with frontotemporal dementia, led us to favor a diagnosis of early-onset AD. A recent neuroimaging study has found that the volumes of frontal, temporal and other brain regions can predict the behavioral and psychological symptoms in AD, and the frontal lobe volume was the most powerful predictor [36]. Therefore, the frontotemporal lobe atrophy of this patient may associate with the early onset of behavioral and psychological symptoms.

There is an overlapping syndrome with diffuse brain structural, metabolic changes, cognitive decline and parkinsonian motor symptoms related to SORL1 gene mutation. Early-onset AD should be considered when a patient initially presenting psychotic symptoms and SORL1 gene mutation is older than the typical age of schizophrenia onset. Our patient was initially misdiagnosed with schizophrenia because of misattribution of the behavioral and psychological symptoms of dementia. We suggest that diagnosis of middle-aged or elderly patients with behavioral and psychological symptoms of dementia as the first presentation should involve detailed cognitive function assessment, brain MRI and PET, and analysis of mutations in AD-associated genes.

## Data Availability

This is a single-patient case report. Data sharing is not applicable to this article as no datasets besides those mentioned in the article were generated or analysed.

## Abbreviations

AD: Alzheimer’s disease
MRI: Magnetic resonance imaging
PET: Positron emission tomography
VLOSLP: Very late-onset schizophrenia-like psychosis
Aβ: Amyloid-beta
MMSE: Mini-mental state exam
MoCA: Montreal cognitive assessment
ADL: Activity of daily living
GMV: Grey matter volume
